# P190B RhoGAP has pro-tumorigenic functions during MMTV-Neu mammary tumorigenesis and metastasis

**DOI:** 10.1186/bcr2643

**Published:** 2010-09-22

**Authors:** Peter R McHenry, James C Sears, Matthew P Herrick, Peggy Chang, Brandy M Heckman-Stoddard, Megan Rybarczyk, Lewis A Chodosh, Edward J Gunther, Susan G Hilsenbeck, Jeffrey M Rosen, Tracy Vargo-Gogola

**Affiliations:** 1 Department of Biochemistry and Molecular Biology, Indiana University School of Medicine, 1234 Notre Dame Ave, South Bend, IN 46617, USA; 2 Division of Cancer Prevention, National Cancer Institute, 6120 Executive Blvd, Bethesda, MD 20892, USA; 3 Department of Molecular and Cellular Biology, Baylor College of Medicine, One Baylor Plaza, Houston, Texas 77030, USA; 4 Department of Cancer Biology, University of Pennsylvania, 421 Curie Blvd, Philadelphia, PA 19104, USA; 5 Department of Medicine, Pennsylvania State Milton S. Hershey Medical Center, 500 University Dr., Hershey, PA 17033, USA; 6 Lester and Sue Smith Breast Center and Department of Medicine, Baylor College of Medicine, One Baylor Plaza, Houston, TX 77030, USA

## Abstract

**Introduction:**

Rho GTPases are overexpressed and hyperactivated in human breast cancers. Deficiency of p190B RhoGAP, a major inhibitor of the Rho GTPases, inhibits mouse mammary tumor virus long terminal repeat (MMTV)-Neu/ErbB2 mammary tumor formation and progression in part through effects within the stromal environment, suggesting that p190B function is pro-tumorigenic. To further investigate the potential pro-tumorigenic actions of p190B, we examined the effects of exogenous p190B expression within the mammary epithelium on MMTV-Neu tumor formation and progression.

**Methods:**

Tetracycline (tet)-regulatable p190B transgenic mice were bred to MMTV-Neu mice, and the effects of exogenous p190B expression on tumor latency, multiplicity, growth rates, angiogenesis, and metastasis were examined. The effects of exogenous p190B expression on cell-matrix adhesion and invasion were tested using non-transformed primary mammary epithelial cells (MECs). Rho GTPase activity, oxidative stress as an indicator of reactive oxygen species (ROS) production, and downstream signaling pathways were analyzed.

**Results:**

Altered p190B expression resulted in a two-fold increase in tumor multiplicity and a three-fold increase in metastases compared to control mice indicating that exogenous p190B expression in the mammary epithelium promotes MMTV-Neu mammary tumor formation and progression. Interestingly, non-transformed primary MECs expressing exogenous p190B displayed increased adhesion to laminin and type IV collagen and formed invasive structures in a three-dimensional culture assay. Ras related C3 botulinum toxin 1 (Rac1)-GTP levels were elevated in p190B transgenic tumors whereas Ras homologous A (RhoA) and cell division cycle 42 (Cdc42)-GTP levels were not significantly altered. Rac1 activity affects production of ROS, which regulate transformation, metastasis, and oxidative stress. Protein carbonylation, which is indicative of oxidative stress, was elevated 1.75-fold in p190B transgenic tumors as compared to control tumors suggesting that exogenous p190B expression may affect Rac1-dependent ROS production.

**Conclusions:**

These studies indicate that paradoxically, p190B RhoGAP, a major inhibitor of the Rho GTPases *in vitro*, has pro-tumorigenic functions that enhance MMTV-Neu induced mammary tumor formation and metastasis. Furthermore, exogenous p190B expression enhances cell adhesion and invasion, which may facilitate metastasis. Rac1 activity and oxidative stress are elevated in tumors expressing exogenous p190B suggesting that p190B may promote tumorigenesis through a Rac1/ROS dependent mechanism.

## Introduction

Rho GTPases including RhoA, Rac1, and Cdc42 integrate extracellular signals to affect a variety of cellular processes such as proliferation, survival, and motility. With their pleiotropic actions it is not surprising that Rho GTPases have been implicated in nearly every stage of breast tumor formation and progression [1]. Numerous *in vitro *and relatively limited *in vivo *studies have demonstrated that Rho GTPases affect transformation, angiogenesis, invasion, and metastasis [2,3]. RhoA, Rac1, Cdc42, and other family members are overexpressed and hyperactivated in early and late stage human breast tumors, and elevated RhoA and Rac1 expression correlates with breast cancer progression [4,5]. Thus, Rho GTPases are likely critical for human breast tumor formation and metastatic progression (for review see [6]).

Rho GTPase activity is tightly regulated both spatially and temporally in cells by positive regulators, guanine nucleotide exchange factors (GEFs), and negative regulators, GTPase activating proteins (GAPs) and guanine nucleotide dissociation inhibitors (GDIs). Paradoxically, some GDIs and GAPs, which are classically thought to be negative regulators of the GTPases, have oncogenic functions [7,8]. Furthermore, Rho GTPase activities vary in different transformed cell lines, and it has been suggested that the expression and activation profile of the Rho GTPases may depend on the selective pressures within a particular tumor [2]. Thus, how the complex regulation of the Rho signaling network is altered during the stochastic process of tumor formation and progression *in vivo *is not well understood. It is clear that there is a need for *in vivo *studies investigating the role of this multifaceted signaling network during mammary tumorigenesis, and this is what we aimed to do in our studies investigating the effects of loss and gain of p190B function on MMTV-Neu mammary tumorigenesis.

P190B RhoGAP is a major regulator of Rho GTPases [9]. In *in vitro *assays, p190B has GAP activity against RhoA, Rac1, and Cdc42 [10]. P190B regulates Rac1 through direct interactions and RhoA indirectly [11]. P190B is also expressed in the developing embryonic mammary anlagen and postnatal mammary gland [12]. Knockout of p190B perturbs embryonic mammary gland development and prevents postnatal ductal morphogenesis indicating that p190B is essential for mammary gland development [12,13]. We hypothesized that if p190B is critical for mammary gland development it might also play an important role during mammary tumorigenesis.

To investigate the role of p190B in mammary tumorigenesis we previously examined the effects of p190B deficiency on MMTV-Neu induced tumor formation and progression [8]. Loss of one allele of p190B delayed tumor onset and reduced tumor penetrance. Furthermore, tumor angiogenesis was inhibited by p190B heterozygosity. Reciprocal transplantation assays demonstrated that p190B expression in the stroma and/or vasculature was likely critical for the angiogenic switch needed to promote progression of preneoplastic lesions. In addition to its effects on tumor formation, p190B deficiency also inhibited lung metastasis when mice with similar tumor burdens as wildtype mice were compared. Thus, p190B plays an important role in the stromal compartment during tumorigenesis and metastatic progression. However, because p190B was deficient in both the stroma and epithelium it was difficult to assess whether p190B expression in the epithelium was also critical for tumor formation and progression. To address this question we utilized tet-regulatable p190B transgenic mice in which p190B is inducibly expressed in the mammary epithelium in response to doxycycline treatment and activation of MMTV-driven reverse tetracycline transactivator [14]. Here we show that p190B expression in the mammary epithelium of MMTV-Neu transgenic mice increases tumor multiplicity and metastasis. Furthermore, non-transformed primary MECs isolated from tet-regulatable p190B transgenic mice display increased adhesion to basement membrane proteins and give rise to invasive structures in a 3 D morphogenesis assay. Thus, p190B regulates adhesion and invasion, which may facilitate metastasis. P190B deficiency in MMTV-Neu tumors decreased Rac1 activity. Conversely, Rac1 activity levels were elevated in p190B transgenic tumors. Regulation of ROS-mediated signaling by Rac1 has been implicated in oxidative stress, transformation, metastasis, and cell survival [15]. Intriguingly, protein carbonylation, an indicator of oxidative stress [16], was elevated suggesting that ROS may be increased downstream of Rac1 in tumors expressing exogenous p190B. We propose that p190B may enhance transformation and metastasis through a Rac1/ROS dependent mechanism. These results, together with our previous studies [8], indicate that p190B may promote mammary tumorigenesis in part by affecting Rac1-mediated signaling.

## Materials and methods

### Mouse strains and tumor analysis

Tet-O-p190B-IRES-luciferase/MMTV-Neu bigenic mice were bred to MMTV-rtTA/MMTV-Neu transgenic mice to obtain trigenic Tet-O-p190B-IRES-luciferase/MMTV-rtTA/MMTV-Neu (*n *= 27), and control Tet-O-p190B-IRES-luciferase/MMTV-Neu (*n *= 19) and MMTV-rtTA/MMTV-Neu (*n *= 28) bigenic mice. All mice were maintained on an FVB background. Genotypes were confirmed by PCR as previously described [8,14,17,18]. Beginning at 10 weeks of age, all mice were fed doxycycline-containing chow (2g/kg ad libitum) for the duration of the study. Mice were palpated weekly beginning at five months of age and once tumors were detected, tumor diameter was measured twice weekly with calipers. Mice were euthanized when tumors reached 1 cm in diameter or when tumor burden reached approximately 10% of body weight in the case of multiple tumors. The Institutional Animal Care and Use Committee of Baylor College of Medicine approved animal protocols, and studies were conducted in accordance with the provisions of the Guide for the Care and Use of Laboratory Animals and the Animal Welfare Act. Kaplan-Meier analysis was done to determine tumor-free survival, and log-rank test analysis was used to determine if differences between the three groups were statistically significant. A Mann-Whitney test was used to determine statistical significance of tumor multiplicity results. An unpaired T-test was used to evaluate the statistical significance of the tumor distribution data. Error bars represent standard error of the mean.

### Tissue preparation and analysis

Mice were given an intraperitoneal injection of BrdU (100 mg/kg) two hours prior to euthanization. Tumors were dissected and divided into pieces that were either fixed in 4% paraformaldehyde and paraffin embedded or flash frozen. Lungs were also collected, fixed, and paraffin embedded. Mammary gland pairs 2/3 and 4 were dissected, fixed, and wholemount stained as previously described [14]. Luciferase assays to detect transgene expression in tumor lysates were done as previously described [14]. Five μm serial sections of tumor and lungs were cut for subsequent histological and immunostaining analysis. Hematoxylin and eosin (H&E) staining was done using standard protocols. To quantify metastasis, the number of metastatic lesions was counted in H&E stained serial sections of the entire lung from *n *= 10 mice per genotype, and ANOVA was used to evaluate statistical significance of these results. Error bars represent standard error of the mean.

### Rho GTPase activation assays

Activities of Rho family GTPases were measured using luminescent (RhoA and Rac1) or colorimetric (Cdc42) G-LISA assays (Cytoskeleton, Denver, CO, USA) according to the manufacturer's instructions. Frozen tumors from p190B (*n *= 11) and rtTA/Neu mice (*n *= 11) were pulverized using a mortar and pestle and homogenized in lysis buffer containing protease inhibitors (Cytoskeleton) using a needle and syringe. The lysates were centrifuged for two minutes/4°C/14,000 × *g*, and the clarified lysates were aliquoted, snap frozen in liquid nitrogen, and stored at -80°C. Protein concentration was determined using the protein assay reagent provided. Immediately before the assays, lysates were thawed and protein concentrations were equalized to 2.0 mg/ml in lysis buffer. For the luminescent assays, SuperSignal West Dura chemiluminescent substrate (Thermo Scientific, Rockford, IL, USA) was substituted for the kit substrate, and a Kodak Gel Logic 1500 digital imaging system and Kodak 1 D software (Carestream Health, New Haven, CT, USA) were used to determine luminescent intensity. For the colorimetric assay, a SpectraMax Plus 384 spectrophotometer and SoftMax Pro software (Molecular Devices, Sunnyvale, CA, USA) were used to determine absorbance. A Mann-Whitney test was used to determine statistical significance of the data. Error bars represent the standard error of the mean.

### Western blotting and luciferase assays

Protein lysates prepared for the G-LISA assays as described above were utilized for Western blotting. Sodium fluoride (20 mM) and sodium vanadate (1 mM) phosphatase inhibitors were added to the samples and equal amounts of protein from each of the 11 samples were pooled prior to analysis. The pooled lysates were electrophoresed on 6%, 8%, 12%, or 15% SDS-polyacrylamide gels, and transferred to PVDF membrane (Millipore, Bedford, MA, USA). Membranes were blocked in 5% milk/TBS-Tween 20 followed by incubation with the following antibodies and concentrations: p190B 1:250 (BD Bioscience, San Jose, CA, USA), ErbB2/Neu 1:1000 (Neomarkers, Fremont, CA, USA), ErbB3 1:1000 (Santa Cruz Biotechnology, Inc., Santa Cruz, CA, USA), ERK-1/2 1:1000 (Cell Signaling, Danvers, MA, USA), phospho-ERK-1/2 1:2000 (Cell Signaling), PAK-1/2/3 1:1000 (Cell Signaling), phospho-PAK1/2 1:1000 (Cell Signaling), β-actin 1:2000 (Sigma, St. Louis, MO, USA), MMP-3 1:200 (Santa Cruz), ROCK-II 1:200 (AnaSpec, Fremont, CA, USA), pROCK/Thr396 1:200 (AnaSpec), AKT 1:1000 (Cell Signaling), pAKT/Ser473 1:1000 (Cell Signaling). Blots were incubated with Pierce West Dura or West Femto Supersignal chemiluminescence reagent (Thermo Scientific, Rockford, IL, USA), and imaged using Kodak Gel Logic 1500 system. Densitometric analysis of Western blots was done using the Kodak Gel Logic system. Data were normalized to β-actin and represent fold change compared to control samples.

### RNA isolation and RT PCR

Frozen tumors were cooled with liquid nitrogen and pulverized with a mortar and pestle. RNA was isolated from the powder using Trizol reagent (Invitrogen, Carlsbad, CA, USA) according to the manufacturer's recommendations. The RNA was treated with DNase I and further purified using an RNeasy RNA purification column (Qiagen, Valencia, CA, USA). One μg RNA was converted to cDNA using a high capacity RNA-to-cDNA kit (Applied Biosystems, Carlsbad, CA, USA). As a negative control, samples were prepared without reverse transcriptase. GAPDH was amplified as an RT-PCR control gene. cDNA was PCR amplified using the same primers used for genotyping: *5'-CCT CAA AAA GTC ATG GGG AAC GGA GC-3' and 5'-CGC TGA CAC GGT AGA GTC CTT CGG-3'*. Reaction conditions were: 94°C for 3 minutes, then 30 cycles of 94°C for 30 seconds, 60°C for 45 seconds, and 72°C for 45 sseconds, followed by 72°C for 5 minutes.

### Oxidative stress assay

The OxyBlot assay (Millipore) was used according to the manufacturer's instructions. Equal amounts of protein from 11 animals per genotype were electrophoresed on a 4 to 15% gradient polyacrylamide SDS gel (Bio-Rad, Hercules, CA, USA). West Dura chemiluminescence reagent was used to develop the blot. Images were captured and data were quantified using the Kodak Gel Logic system. Densitometry values represent fold change relative to the control, and samples were normalized to β-actin.

### Immunostaining and quantification of staining

Immunohistochemistry was done as previously reported [14] using biotin-conjugated BrdU 1:10 (BD Pharmingen, 550803, San Diego, CA, USA), cleaved caspase-3 1:400 (Cell Signaling), and CD31 1:500 (Abcam, Cambridge, MA, USA) in M.O.M block (Vector Labs, Burlingame, CA, USA). F4/80 1:50 (Abcam) immunofluorescent staining was done as previously described [19]. Two independent observers blinded to the experimental groups assessed vascular density by counting the number of vessels, *n *= 13, rtTA/Neu and *n *= 11, p190B, an average of eight 400× fields were counted per tumor, and proliferation by counting the number of BrdU positive cells in a minimum of five 400× fields from each tumor. F4/80 staining was quantified by counting a minimum of three 400× fields from each tumor. Cleaved caspase-3 staining was quantified by counting five 400× fields per tumor representing approximately 10,000 cells, *n *= 10 tumors per genotype. Images were captured using a Zeiss Axioimager epifluorescence microscope (Carl Zeiss, Inc., Baden-Werttemburg, Oberkochen, Germany).

### Isolation, culture, and immunostaining of primary MECs

MECs were isolated from mammary glands (MGs) of paired 7 to 10 week-old MMTV-rtTA and tetO-p190B/MMTV-rtTA mice. MGs were resected and placed in ice-cold wash buffer (DMEM/F12 medium supplemented with 5% FBS, 50 μg/mL gentamicin, and 2.5 μg/mL amphotericin B). MGs were minced and incubated in digestion medium (DMEM/F12 medium supplemented with 50 μg/mL gentamicin, 2.5 μg/mL amphotericin B, 2 mg/mL collagenase, and 100 U/mL hyaluronidase; 10 mL/g of tissue) shaking at 200 rpm at 37°C for one hour. The digest was diluted with wash buffer and centrifuged at 450 *g*/4°C/5 minutes. The fat and aqueous layers were aspirated and discarded, and the cell pellet was washed three times in ice-cold wash buffer, repeating the centrifugation conditions above. The cell pellet was washed once in ice-cold PBS then incubated with 0.05% trypsin-EDTA shaking at 200 rpm at 37°C for 10 minutes. Following trypsin digest, ice-cold wash buffer was added, and the cell suspension was filtered through a 70 μm cell strainer. The strainer was rinsed once with wash buffer to increase cell yield. The cells were pelleted as before and resuspended in growth medium (Clonetics MEGM complete medium, Lonza, Basel, Switzerland) and seeded at a density of 2.5 × 10^6 ^cells per well in a six-well plate pre-incubated with 1 mL/well growth medium containing 2 mg/mL fetuin and grown overnight. Non-adherent cells were removed by two washes with PBS and discarded. Adherent cells were detached with 0.05% trypsin-EDTA, diluted in growth medium, pelleted, and resuspended in ice-cold growth factor-reduced Matrigel (BD Biosciences, San Jose, CA, USA) to a density of 7,500 cells per 40 μl (per well of eight-well chamber slide). Matrigel containing MECs was pipetted onto chamber slides without contacting walls, and the slides were incubated at 37°C for 15 minutes. Growth medium containing 2% Matrigel and 2 μg/mL doxycycline was added to the chamber wells, and the cells were grown at 37°C/5% CO_2_/dark/humidified for seven days. The medium was changed every two to three days. For invasion assays, one hundred structures were analyzed per experiment (*n *= 4 experiments), t tests were used to compare means, and error bars represent standard error of the mean. For the inhibitor studies, ERK inhibitor (EMD Biosciences, San Diego, CA, USA) was used at 20 μM and NSC23766 (Tocris Bioscience, Bristol, UK) was used at 25 μM. Inhibitors were diluted in DMSO and added along with doxycycline (2 μg/ml) when the cells were seeded. Media was changed and inhibitors were added every three days for one week. Cultures were fixed, stained and analyzed as described below. One hundred structures were analyzed per experiment and the experiments were repeated three times. T-tests were done to determine statistical significance of the data and error bars represent standard error of the mean.

Immunofluorescence was performed according to Debnath *et al*., using 2% paraformaldehyde as the fixative [20]. The secondary blocking step was omitted. Primary antibodies, αtubulin 1:200 (Sigma Immunochemicals, St. Louis, MO, USA), p190B 1:200 (BD Transduction Labs), pERM 1:200 (Cell Signaling), βtubulin 1:200 (Sigma Immunochemicals) were applied in primary blocking solutions overnight at room temperature. Secondary antibodies, goat anti-rabbit Alexafluor 488 and anti-mouse Alexafluor 594, were used at 1:200. The slides were mounted with Vectashield mounting medium containing DAPI (Vector Laboratories, Servion, Switzerland), sealed with nail polish, and stored at room temperature.

### Adhesion assays

Primary MECs were isolated from p190B transgenic and rtTA control mice that had been fed Dox chow (2 g/kg) for three days to induce transgene expression and to control for off-target effects of Dox, respectively. MECs from three mature virgin mice per genotype were isolated as described for invasion assays, except that the washes following collagenase digestion were performed at 600 × *g*/4°C/1 minute and repeated three to six times to remove all erythrocytes. Isolated MECs were pooled and plated on fetuin-coated plastic with growth medium (DMEM/F12 supplemented with 5 μg/ml insulin, 1 μg/mL hydrocortisone, 5 ng/mL epidermal growth factor (EGF), 50 μg/ml gentamicin, 2.5 μg/mL amphotericin B, and 5% FBS) and treated overnight with Dox (2 μg/ml) to maintain transgene expression. Alternatively, cells were frozen for later use in 90% FBS/10% DMSO and seeded on fetuin-coated plastic upon thawing. Cells were trypsinized, washed with serum-containing medium to inactivate the trypsin, and transferred into 96-well plates pre-coated with 15 μg/cm^2 ^of type I collagen, type IV collagen, laminin, or fibronectin. After incubation at 37°C (two hours for collagen I, IV, and fibronectin; three hours for laminin), non-adherent cells were washed off with PBS. Remaining cells were incubated with Cell Titer 96 Aqueous One Solution (Promega, Madison, WI, USA) for one hour at 37°C, and absorbance at 490 nm was measured with a SpectraMax Plus 384 spectrophotometer and SoftMax Pro software (Molecular Devices, Sunnyvale, CA, USA). Experiments were performed in triplicate and repeated three times. Means were compared using a t-test, and error bars represent standard error of the mean.

## Results

### P190B transgene expression in the mammary epithelium of MMTV-Neu mice increases tumor multiplicity

Previously we demonstrated that loss of one allele of p190B markedly delayed MMTV-Neu tumor latency and penetrance [8]. Because p190B was deficient in both the epithelial and stromal compartments of the mammary gland it was difficult to distinguish whether loss of p190B in the epithelium impacted tumor initiation. To determine the effects of epithelial expression of p190B on mammary tumorigenesis we bred tetracycline-regulatable p190B transgenic mice to MMTV-Neu mice. Two different control groups, tet-O-p190B-IRES-luciferase/MMTV-Neu (*n *= 19) and MMTV-rtTA/MMTV-Neu (*n *= 28) bigenic mice, were compared to exogenous p190B expressing, tet-O-p190B-IRES-luciferase/MMTV-rtTA/MMTV-Neu (*n *=27) trigenic female mice. For simplicity we refer to the tet-O-p190B/MMTV-Neu control group as tetO/Neu, the MMTV-rtTA/MMTV-Neu control group as rtTA/Neu, and the p190B/MMTV-rtTA/MMTV-Neu experimental group as p190B.

**Figure 1:**
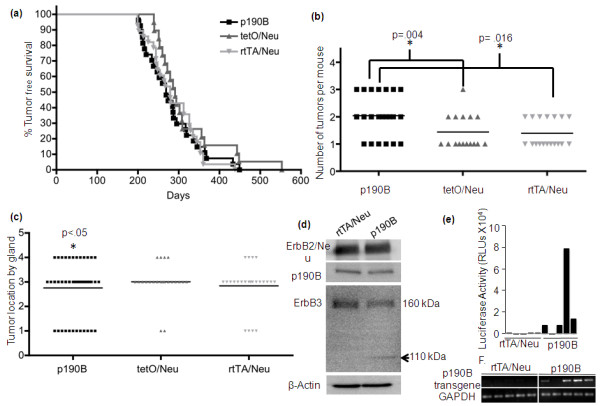
**Exogenous expression of p190B increases tumor multiplicity**. **(a) **Kaplan-Meier tumor-free survival curve of rtTA/Neu, tetO/Neu, and p190B transgenic mice reveals that p190B does not affect MMTV-Neu tumor latency. The median time to tumor onset was approximately 275 days for each of the three groups. Log-rank test analysis showed no significant differences between the groups (*P *> 0.05). **(b) **P190B transgenic mice developed an average of two tumors whereas control mice developed an average of one tumor per mouse indicating that exogenous p190B expression increases tumor multiplicity. A Mann-Whitney test revealed statistically significant differences in tumor multiplicity between the groups. **(c) **Tumor location by gland is graphed demonstrating statistically significant increased tumor formation in mammary gland pairs 1, 4, and 2/3 in the p190B transgenic mice as compared to rtTA/Neu control mice (*P *< 0.05) and in mammary gland pairs one and four compared to tetO/Neu control mice (*P *< 0.05). **(d) **Western blotting to detect expression of ErbB2/Neu, p190B, and ErbB3 in rtTA/Neu and p190B transgenic tumors. Equal amounts of protein were pooled from 11 mice per genotype. Similar levels of ErbB2 and p190B were detected in the two groups. Arrow indicates a lower molecular weight form of ErbB3 that was detected concomitant with a decrease in total protein in p190B transgenic tumor lysates. β-actin is shown as a loading control. **(e) **Luciferase activity is graphed indicating that the tetO-p190B-IRES-luciferase transgene is expressed in the majority of p190B tumors. Luciferase activity is never detected in control tumors. **(f) **RT-PCR on cDNA prepared from exogenous p190B expressing and rtTA/Neu control tumors shows mRNA expression of the human p190B transgene in p190B transgenic, but not rtTA/Neu control tumors. RT-PCR for GAPDH is shown as a positive control and samples prepared without RT were negative (data not shown).

We have previously shown that exogenous p190B expression during virgin development delayed ductal outgrowth, increased side branching, and resulted in disorganization of the ductal tree [14]. To ensure that mammary gland developmental defects would not impact tumor formation and progression, p190B transgene expression was induced beginning at 10 weeks of age. Mice were fed doxycycline (2 g/kg Dox containing chow *ad libitum *for the duration of the study) to induce transgene expression in the trigenic mice and to control for any effects of doxycycline on mammary tumorigenesis and progression in the bigenic control groups. Beginning at five months of age, mice were palpated weekly to detect tumors, and the age of onset and location of the tumor were recorded. In contrast to p190B haploinsufficiency, which markedly delayed tumor onset, p190B transgene expression did not affect tumor latency (Figure 1a). The median time to tumor onset for each group was approximately 280 days as determined by Kaplan-Meier analysis. Although targeted expression of p190B in the epithelium did not alter the timing of tumor onset, it significantly increased tumor multiplicity (Figure 1b). P190B transgenic mice developed an average of two tumors per mouse whereas single tumors were detected in the majority of the control mice (p190B vs. tetO/Neu, *P *= 0.004, p190B vs. rtTA/Neu, *P *= 0.016). The distribution of tumors also differed significantly between the control and p190B transgenic mice. Compared to rtTA/Neu mice, p190B mice formed more mammary tumors in the number 1 and 4 (44 +/- 10 vs. 14 +/- 7 percent of mice, *P *= 0.013) and 2/3 (93 +/- 5 vs. 61 +/- 9 percent of mice, *P *= 0.005) mammary gland pairs (Figure 1c). Compared to tetO/Neu mice, p190B mice formed more mammary tumors in number 1 (44+/-10 vs. 11+/-7 percent of mice, *P *= 0.013), and number 4 (44 +/- 10 vs. 16 +/- 9 percent of mice, *P *= 0.042) mammary gland pairs. P190B and tetO/Neu mice formed similar numbers of tumors in the 2/3 (93 +/- 5 vs. 95 +/- 5 percent of mice, *P *> 0.05) mammary gland pairs. Because tumor latency, multiplicity, and distribution in the tetO/Neu mice were similar to rtTA/Neu mice, we compared Dox-treated p190B and rtTA/Neu mice for all subsequent analyses.

To assess whether exogenous p190B expression may increase tumor multiplicity by affecting expression of the Neu transgene Western blotting was performed on pooled lysates prepared from rtTA/Neu and p190B tumors (*n *=11 tumors per genotype) (Figure 1d). Because endogenous ErbB3 has been shown to be important for MMTV-Neu induced tumorigenesis and survival signaling through direct affects on AKT [21], we also examined ErbB3 expression. Whereas ErbB2 expression levels were similar, we detected a slight decrease in expression of full length ErbB3 and the appearance of a smaller band suggesting that ErbB3 may be proteolytically cleaved in p190B tumors. To verify transgene expression and to determine the extent of exogenous p190B expression we performed luciferase assays on tumor lysates from the p190B transgenic mice and Western blotting to detect p190B in the pooled tumor lysates. Luciferase was expressed in 7/8 tumors from the p190B mice whereas, as expected, none of 10 tumors from control mice expressed luciferase (Figure 1e and data not shown). We also performed RT-PCR on cDNA prepared from rtTA/Neu and p190B tumors using primers that specifically amplify the human p190B transgene, but not the endogenous p190B. Consistent with the luciferase data, 4/5 p190B tumors expressed the human p190B transgene mRNA, which was not detectable in any of the rtTA/Neu control tumors (Figure 1f). Despite expression of luciferase and human p190B mRNA, which indicated that the tetO-p190B-IRES-luciferase transgene was active in the majority of tumors, overexpression of p190B was not detectable by Western analysis (Figure 1d). This was not surprising as we were previously unable to detect overexpression of the p190B transgene in the developing mammary glands of these mice by Western blotting [14]. However, low levels of p190B transgene expression in the developing mammary gland rapidly and markedly altered terminal end bud architecture and ductal morphogenesis [14]. In addition, the majority, but not all of the tumors expressed the p190B transgene, and thus, by pooling tumor lysates from 11 mice we may have diminished any small increases in p190B protein expression detectable by Western blot. These results indicate that p190B is not overexpressed at non-physiological levels in this model and that even small or potentially transient increases in p190B expression in the mammary epithelium affects MMTV-Neu induced mammary tumorigenesis.

### P190B and rtTA/Neu tumors have similar histologies, proliferation, and apoptosis rates

**Figure 2:**
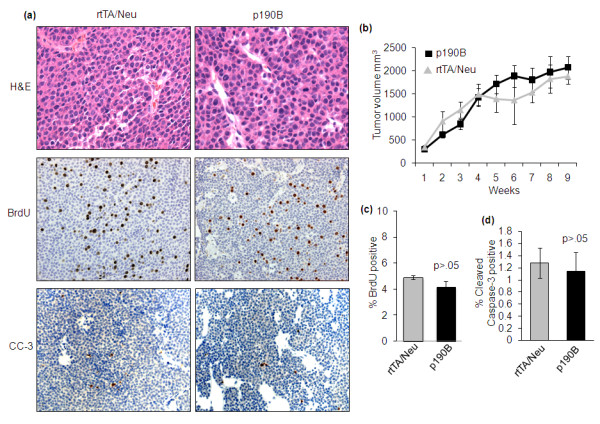
**P190B transgene expression does not affect tumor differentiation, proliferation, growth rates, or apoptosis**. **(a) **H&E stained tumor sections (400× magnification) demonstrate that p190B does not alter differentiation of MMTV-Neu tumors. Representative images of immunostaining to detect BrdU incorporation and cleaved caspase 3 (200× magnification) in 5 μm tumor sections from rtTA/Neu and p190B mice are shown. **(b) **Growth curves of p190B and rtTA/Neu control tumors are graphed. No statistical differences were detected in tumor growth rates (t test, *P *> 0.05). Error bars represent the standard error of the mean. **(c) **Quantification of BrdU incorporation shows that exogenous p190B expression does not affect tumor cell proliferation rates (t test, *P *> 0.05). **(d) **Cleaved caspase 3 (CC3) immunostaining is quantified and demonstrates that p190B transgene expression does not affect tumor cell survival (t test, *P *> 0.05). Error bars represent the standard error of the mean.

Previously we demonstrated that p190B deficiency did not impact the histology of the MMTV-Neu tumors [8]. Similarly, exogenous expression of p190B did not alter the histology of the tumors, which were classified as poorly differentiated adenocarcinomas typical of those found in MMTV-Neu transgenic mice [22] (Figure 2a). Tumor growth rates were also not statistically different between the groups as determined by analysis of tumor volumes beginning from the time of palpation until the mice were euthanized (Figure 2b). Analysis of BrdU incorporation, a marker of S phase, indicated that proliferation rates were similar in p190B transgenic and control tumors (4.1 +/- 0.13% vs. 4.9 +/- 0.49%, *P *> 0.05, *n *=6 tumors/group, a minimum of 5,000 cells/tumor were counted) (Figure 2a, c). To evaluate whether p190B affects tumor cell survival we quantified the number of cleaved caspase-3 (CC-3) positive cells in tumor sections (five fields per tumor representing approximately 10,000 cells were quantified, *n *=10 tumors per genotype). This analysis did not reveal any differences in apoptosis rates in p190B tumors compared to control tumors (1.14 +/- 0.32% vs. 1.28 +/- 0.25%, *P *> 0.05) (Figure 2a, d). Thus, these data indicate that exogenous p190B expression does not alter differentiation, proliferation, or apoptosis in MMTV-Neu tumors.

### P190B transgene expression does not affect the angiogenic switch

**Figure 3:**
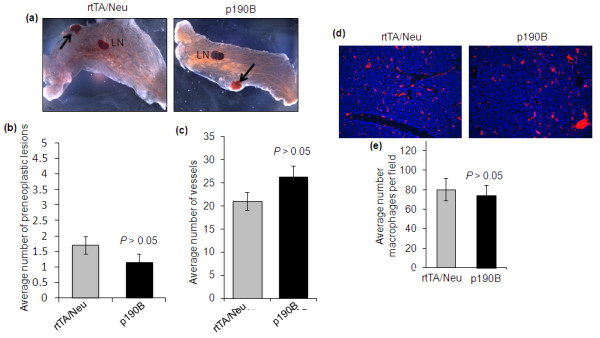
**Exogenous p190B expression does not promote MMTV-Neu induced tumorigenesis by affecting the angiogenic switch**. **(a) **Whole-mount mammary glands from tumor-bearing mice were examined to detect hyperplastic lesions. Representative images are shown with arrows indicating hyperplastic lesions. LN indicates lymph nodes. **(b) **The number of hyperplastic lesions were quantified in tumor-free mammary glands pairs 2/3 and 4 from tumor-bearing mice (*n *=17 rtTA/Neu and *n *=13 p190B mice). No statistical differences were detected (t test, *P *> 0.05). Error bars represent standard error of the mean. **(c) **Quantification of CD31 immunostaining in tumor sections from p190B (*n *=11) and rtTA/Neu (*n *=13) mice demonstrates that p190B does not affect tumor angiogenesis. An average of eight 400× fields were counted per tumor. No statistical differences were detected (t test, *P *> 0.05). Error bars indicate standard errors **(d) **F4/80 immunostaining to detect macrophage infiltration in p190B and rtTA control tumors. F4/80 is shown in red and nuclei are blue. Representative 400× images are shown. **(e) **Quantification of F4/80 immunostaining demonstrates that there is no difference in macrophage infiltration in p190B transgene expressing tumors as compared to rtTA control tumors. Five 400× fields per tumor (*n *=7 per genotype) were quantified. No statistical differences were detected (t test, *P *> 0.05). Error bars indicate the standard error of the mean.

Haploinsufficiency for p190B blocked preneoplastic progression by inhibiting angiogenesis [8]. To determine whether exogenous p190B expression affected tumorigenesis by altering initiation and progression of preneoplastic lesions we quantified the number of microscopic, non-palpable lesions in whole-mounted mammary glands from rtTA/Neu (*n *=17) and p190B (*n *=13) mice. Similar numbers of preneoplastic lesions were detected in both groups (1.2 +/- 0.27 vs. 1.5 +/- 0.24 preneoplastic lesions per gland, *P *> 0.05) (Figure 3a, b). Furthermore, in contrast to p190B deficiency, which inhibited tumor angiogenesis, exogenous p190B expression did not impact the development of tumor vessels. Quantification of CD31 staining revealed that similar numbers of vessels were present in tumors from control and p190B mice (21 +/- 1.9 vs. 26 +/- 2.4, *P *> 0.05) (Figure 3c). These results suggest that exogenous p190B expression in the epithelium does not promote tumor progression by impacting angiogenesis.

Previously we showed that p190B transgene expression in the developing mammary gland increased macrophage infiltration in association with aberrant terminal end bud structures [14]. Macrophages have also been shown to promote mammary tumor formation, invasion, and metastasis [19,23]. To determine whether macrophage infiltration is altered by exogenous p190B expression, tumor sections were immunostained with the macrophage marker F4/80 and the number of positive cells were quantified in a minimum of three fields per tumor (*n *=7 tumors per genotype). This analysis demonstrated that there was no significant difference in macrophage infiltration into the tumors (80 +/- 11 vs. 75 +/- 10, *P *> 0.05) (Figure 3d, e). Thus, macrophage infiltration does not correlate with the increased tumorigenesis detected in p190B transgenic mice. However, these results do not rule out of the possibility of altered activation of macrophages in the tumors expressing exogenous p190B.

### P190B transgene expression increases cell adhesion, invasion and lung metastasis

**Figure 4:**
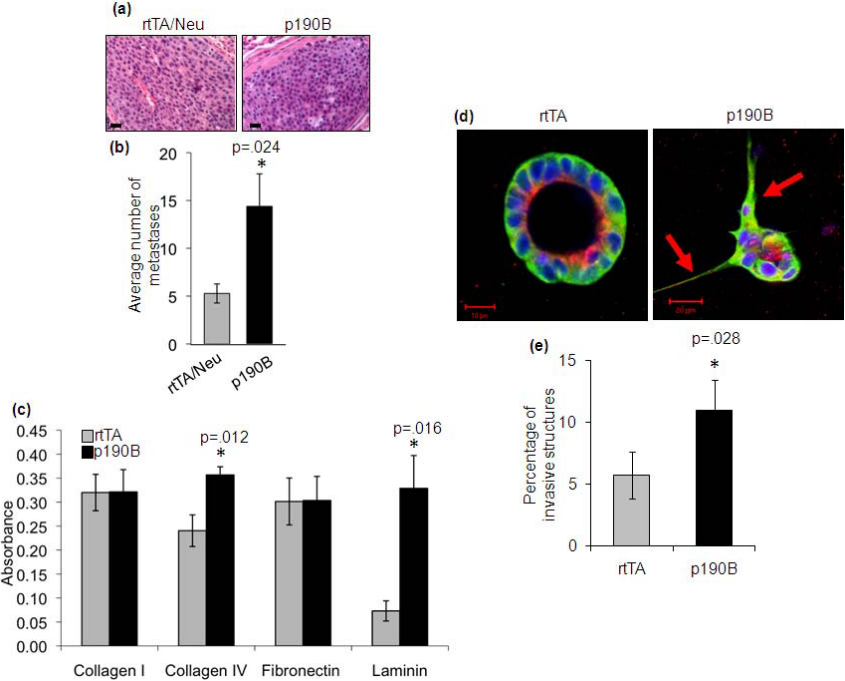
**P190B transgene enhances cell-ECM adhesion, invasion, and metastasis**. **(a) **Exogenous p190B expression does not alter the histopathology of MMTV-Neu lung metastases, which resemble undifferentiated adenocarcinomas that are typical of MMTV-Neu mice. **(b) **Average number of metastases per mouse at tumor burden was determined by counting metastatic lesions in serial sections of the entire lung. rtTA/Neu control mice had similar numbers of metastases as TetO/Neu mice (data not shown). ANOVA was used to determine statistical significance between the groups. Error bars represent standard error of the mean. **(c) **P190B (black bars) and rtTA (gray bars) primary MECs were analyzed for their ability to adhere to ECM proteins. An MTS assay was used as an indirect measurement of the number of adherent cells. Data represent three independent experiments. A t test was used to determine statistical significance and error bars represent standard errors of the mean. **(d) **Exogenous p190B expression promotes invasion as determined by confocal microscopic analysis of p190B and rtTA primary MECs cultured in Matrigel for 12 days and immunostained to detect red = p190B, gree*n *=αtubulin, blue = DNA. Arrows indicate invasive projections. **(e) **The number of invasive structures was quantified (*n *=400 structures per genotype). A t test was used to determine statistical significance, and error bars represent standard error of the mean.

P190B deficiency inhibited primary tumor formation, and metastasis was also significantly reduced when p190B deficient mice were compared to wildtype mice with similar tumor burdens [8]. To assess whether exogenous p190B expression affects metastasis the histopathology and number of metastases were analyzed in serial sections of lungs from tumor-bearing mice. Lung metastases in both control and p190B mice resembled undifferentiated adenocarcinomas, which are typical of MMTV-Neu primary tumors (Figure 4a). Thus, p190B transgene expression did not alter the histopathology of the metastatic lesions. Interestingly, a three-fold increase in the number of lung metastases was detected in p190B transgenic mice (*n *=10, *P *= 0.024) as compared to rtTA/Neu (*n *=10) and tetO/Neu mice (*n *=8) (Figure 4b and data not shown). These data suggest that exogenous p190B expression may promote metastatic progression.

To explore the mechanisms by which p190B may enhance metastasis, we first tested whether exogenous p190B expression affected cell adhesion to extracellular matrix (ECM) proteins. Cellular adhesion to ECM plays an important role in generating forces required during migration and invasion [24]. For these experiments, non-transformed primary MECs were isolated from p190B/MMTV-rtTA mice and control MMTV-rtTA mice lacking the Neu transgene that had been fed Dox chow (2 g/kg) for three days to induce transgene expression. Once in culture, Dox (2 μg/ml) was added to both cell types for the duration of the experiments. P190B transgenic MECs were 1.5-fold (*P *= 0.012) and 4.5-fold (*P *= 0.016) more adherent to the basement membrane proteins type IV collagen and laminin, respectively, as compared to MECs from control mice (Figure 4c). No differences in adhesion to type I collagen or fibronectin were detected.

To assess whether the increased adhesive capacity of p190B transgenic MECs also affected cellular invasion, we quantified the number of invasive structures that formed when the primary MECs were grown in 3 D culture. This analysis demonstrated that exogenous expression of p190B in primary MECs promoted a two-fold increase in invasion (*P *= 0.035, *n *=400 structures per genotype) (Figure 4d, e). Many of the invasive structures displayed abnormal polarity and disrupted lumens, however, these phenotypes were not significantly different between the two genotypes (data not shown). While we cannot rule out the possibility that the increased tumor multiplicity in p190B transgenic mice may have resulted in more metastases, it appears likely that the enhanced adhesive and invasive capacity of p190B transgenic MECs facilitates metastasis of MMTV-Neu induced mammary tumors.

We previously showed that altered p190B expression affected signaling through Rac and ERK in the developing mammary gland and in MMTV-Neu tumors [8,14]. To determine whether the invasive phenotype is dependent on signaling through Rac and/or ERK we treated primary MECs from p190B transgenic and MMTV-rtTA control mice in 3 D culture with Rac NSC23766 (25 μM) and ERK (20 μM) inhibitors and quantified effects on lumen formation and invasion. As seen in Supplemental figure 1 in Additional file 1, neither inhibitor alone blocked the effects of p190B trasgene expression on invasion. In contrast, inhibition of Rac or ERK significantly increased invasion and disrupted lumen formation and polarity in both control MMTV-rtTA and p190B primary MECs indicating that both Rac and ERK activities are important for normal morphogenesis of primary MECs (Supplemental figure 1 in Additional file 1). It is likely that multiple signaling pathways are perturbed downstream of p190B, and the precise mechanisms by which p190B affects invasion of primary MECs remain to be elucidated.

### Rac1 activity is elevated in tumors expressing exogenous p190B

**Figure 5:**
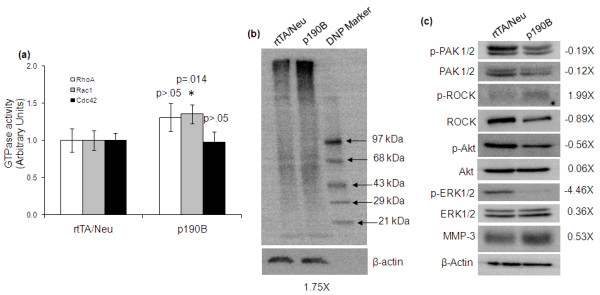
**Rac1 activity and oxidative stress are increased in p190B transgenic tumors**. **(a) **G-LISA assays were used to quantify the levels of active RhoA, Rac1, and Cdc42 in rtTA (*n *=11) and p190B (*n *=11) tumors. A Mann-Whitney rank sum test was used to compare the means, and error bars represent standard error of the mean. Rac1 activity was elevated, however, RhoA and Cdc42 activities were not statistically significantly different between the two groups. **(b) **An OxyBlot assay was performed to detect protein carbonylation as an indirect measure of ROS-mediated oxidative stress. Western blot shows an increase in total carbonylated proteins after dinitrophenylhydrazine (DNP) derivitization in p190B expressing tumor lysates compared to control lysates. Each lane represents equal amounts of protein pooled from 11 tumors per genotype. DNP protein standards were included for size comparison. β-actin is shown as a loading control. Densitometry value represents the fold change in carbonylated proteins in p190B tumors relative to control tumors. Control tumor values were set to 1 and samples were normalized to β-actin. **(c) **Western blotting to detect expression of total and phosphorylated forms of downstream effectors of Rac1. β-actin is shown as a loading control. Each lane represents equal amounts of pooled protein from 11 tumor lysates per genotype. Densitometry values represent the fold change in protein expression in p190B transgenic tumors relative to control tumors. Control tumor values were set to 1 and samples were normalized to β-actin.

P190B has previously been shown to have GAP activity against Rho, Rac, and Cdc42 [10]. More recently, p190B was shown to directly interact with Rac1, but not RhoA or Cdc42. However, RhoA activity was indirectly regulated by p190B [11]. We previously demonstrated that Rac1-GTP levels were reduced in p190B deficient tumors [8]. To determine the effects of exogenous p190B expression on Rho GTPase activities we performed G-LISA assays on tumor lysates from rtTA/Neu and p190B transgenic tumors. This analysis revealed a statistically significant increase in Rac1 activity levels in p190B transgenic tumors (*n *=11, *P *= 0.014) as compared to control tumors (*n *=11) (Figure 5a). No statistical differences were detected in the levels of RhoA or Cdc42 activity. Elevated Rac1 activity promotes transformation and metastasis [25], and deficiency of the RacGEF, Tiam1, inhibits MMTV-Neu tumor formation [26]. Thus, p190B may facilitate tumor formation and progression by enhancing Rac1 activity.

Rac1 regulates production of ROS, and ROS affect tumorigenesis, metastasis, and cell survival [15,27,28]. Because it is not possible to directly measure ROS levels in the tumors *in situ*, we assessed oxidative stress as an indirect measure of altered ROS production. For this we utilized the OxyBlot Western assay to assess protein carbonylation in the pooled tumor lysates (*n *=11 per genotype), which is indicative of oxidative stress [16]. As seen in Figure 5b, protein carbonylation, was increased 1.75-fold in p190B transgenic tumors as compared to control tumors. These results suggest that ROS may be elevated downstream of Rac1 in the tumors expressing exogenous p190B, although additional studies will be required to prove this. Interestingly, expression of MMP-3, which regulates ROS production through a Rac1b-dependent mechanism [15] and is also upregulated by ROS [29], is elevated in p190B trasngenic tumors (Figure 5c).

Next we examined the effects of p190B on downstream signaling within the tumors. For this, Western blotting to detect phosphorylated and total levels of the Rac1 effectors p21 activated kinase (PAK-1/2), the RhoA effector Rho kinase (ROCK), and downstream signaling molecules AKT and ERK was done on the pooled tumor extracts from 11 mice per genotype. Paradoxically, PAK-1/2, ROK, AKT, and ERK expression and/or activity levels are diminished in p190B tumors as compared to rtTA/Neu control tumors (Figure 5c). We have previously shown that sustained exogenous expression of p190B in the developing mammary gland inhibits signaling to these downstream effectors [14]. It is unclear why these signaling molecules are not increased in the p190B transgenic tumors to reflect the elevated Rac1 that was detected in the tumors. Additional studies will be required to dissect the molecular mechanisms underlying the pro-tumorigenic effects of p190B.

## Discussion

The Rho signaling network is perturbed during tumorigenesis [1,30,31], and Rho GTPases are overexpressed and hyperactivated in breast tumors [4,5,32]. *In vitro *studies have shown that this signaling network has pleiotropic functions and can regulate tumor cell proliferation, survival, and migration [3]. Despite the wealth of information available about how Rho GTPases affect cellular processes and signaling *in vitro*, the cellular and molecular mechanisms by which Rho GTPases affect the stochastic process of tumor formation in the complex *in vivo *environment are not well understood. We investigated this by examining the role of p190B RhoGAP, a major inhibitor of Rho GTPases, during MMTV-Neu induced mammary tumor formation and progression.

P190B is required for mammary gland development [12-14], and we hypothesized that it would also be pivotal for mammary tumorigenesis. Previously, we examined the effects of loss of p190B function on MMTV-Neu tumorigenesis and showed that haploinsufficiency for p190B delayed tumor onset and decreased tumor penetrance [8]. P190B was also deficient in the stroma, and transplantation assays indicated that its function in the stroma and/or vasculature was important for the angiogenic switch that is required to promote preneoplastic progression. Although these studies suggested that p190B also played an important role in the epithelium, it was difficult to assess this using the germline p190B knockout mice. We, therefore, turned to tet-regulatable p190B transgenic mice in which p190B is inducibly expressed in the mammary epithelium in response to Dox treatment.

Exogenous p190B expression enhanced MMTV-Neu induced tumorigenesis as indicated by an increase in the number of tumors that formed per mouse, which is consistent with an increase in tumor formation in mammary gland pairs one and four compared to control mice. We did not detect a difference in the number of preneoplastic lesions that formed in the p190B transgenic mice as compared to the control mice, nor did we detect a statistically significant difference in tumor angiogenesis. These data indicate that p190B expression in the epithelium does not facilitate tumor formation by affecting the angiogenic switch. Furthermore, these results are consistent with our data demonstrating that deficient expression of p190B in the stroma and/or vasculature affected progression of preneoplastic lesions in MMTV-Neu mice [8].

Similar to the effects of p190B deficiency in MMTV-Neu tumors [8], p190B transgene expression also had no impact on tumor growth, proliferation rates, or apoptosis as demonstrated by determination of tumor volumes, and quantification of BrdU incorporation and cleaved caspase-3 staining, respectively. Thus, p190B is not likely promoting tumor formation by enhancing proliferation or tumor cell survival.

In addition to promoting primary tumor formation, exogenous p190B expression also increased metastasis. Non-transformed p190B transgenic primary MECs exhibited increased adhesion to basement membrane proteins and formed invasive structures in a 3 D culture morphogenesis assay. These experiments demonstrated that even in the absence of an oncogenic stimulus, p190B promotes cellular invasion. We propose that these phenotypes contributed to the increase in metastasis in the p190B transgenic MMTV-Neu mice. However, we cannot rule out the possibility that the increase in tumor burden due to the presence of multiple tumors in the majority of p190B transgenic mice also impacted metastasis.

We attempted to elucidate the molecular mechanisms by which p190B facilitates invasion in primary non-transformed MECs by testing whether inhibition of Rac or ERK was sufficient to block the invasive phenotype promoted by exogenous p190B expression. However, inhibition of either Rac or ERK throughout MEC morphogenesis significantly perturbed polarity and lumen formation and enhanced invasion regardless of p190B expression. Dominant negative Rac1 expression has previously been shown to disrupt polarity and lumen formation in primary MECs [33] so it was not surprising that Rac inhibition promoted these phenotypes. P190B transgene expression in the developing mammary gland [14] and in MMTV-Neu tumors inhibited ERK activity, and it is possible that inhibition of ERK activity contributes to the invasive phenotype of p190B transgenic mammary acini.

Initially we were surprised that ERK inhibition dramatically altered lumen formation and promoted invasion in the majority of mammary acini in 3 D culture as several studies have demonstrated that sustained or elevated ERK activity promotes MEC transformation and invasion [34-36]. In addition, elevated ERK activity has been linked to poor prognosis in breast cancer [37]. Paradoxically, a number of other studies have demonstrated that ERK signaling can have opposite phenotypic effects in a cell type specific manner depending on the level, duration, and cellular localization of the signal [38]. Furthermore, elevated ERK activity has also been linked to better prognosis in several studies of breast cancer patients [39-41]. The seemingly paradoxical impact of ERK activation on mammary transformation and tumorigenesis may be dependent on the specific genetic alterations within individual breast tumors or cell lines, and the role of ERK activation should be considered within these specific contexts.

To further explore the molecular mechanisms underlying the pro-tumorigenic actions of p190B in MMTV-Neu tumors, we examined the activity levels of RhoA, Rac1, and Cdc42 in the tumors. We previously demonstrated that loss of p190B function in MMTV-Neu tumors leads to decreased Rac1 activity, and now conversely, we show that exogenous p190B expression results in elevated Rac1 activation. Similar to the studies in the p190B deficient mice, we did not detect statistically significant differences in RhoA or Cdc42 activities. However, we cannot rule out the possibilities that activity of other GTPases may be altered or that RhoA and Cdc42 are transiently regulated.

Rac1 has been implicated in transformation and metastasis [25,42,43]. Deficiency of the Rac GEF Tiam1 in MMTV-Neu mice delayed tumor onset and decreased metastasis suggesting that Rac1 activity plays a causal role in MMTV-Neu induced tumor formation and progression [26]. Taken together, these studies have lead us to hypothesize that Rac1 activity may be regulated by p190B during MMTV-Neu mammary tumorigenesis and that p190B may promote transformation and metastatic progression via a Rac1 dependent mechanism. Additional studies will be required to demonstrate whether p190B promotes tumorigenesis via a Rac-dependent mechanism.

A major question raised by our studies is why loss and gain of p190B function, which would be expected to increase and decrease GTPase activity, respectively, has the opposite effect on Rac1 activity? High GTPase activity and rapid turnover in the presence of elevated GAP expression seem to be contradictory. Computational modeling has suggested that although GAPs reduce GTPase activity, the GTPase turnover rate increases proportionally with increasing GAP concentration, and ultimately, the balance between GAP and GEF concentrations is critical for high GTPase activity [44]. In addition, GTPase cycling rates in fast cycling mutants increase the transforming capacity of the Rho GTPases [45,46]. It is possible that through direct interactions, p190B enhances the cycling rate of Rac1, thereby promoting elevated Rac1 activity. P190B may also act as a scaffold to bring together a large signaling complex at the membrane, and thus, exogenous p190B expression may increase Rac1 membrane localization and subsequent activity.

Another question raised by these studies is if Rac1 activity is elevated in p190B transgenic tumors, why is signaling to the downstream effectors AKT and ERK diminished? It is important to note that the Western analysis represents a fixed time point in late stage tumors that may not be indicative of the early changes in signaling that are responsible for tumor initiation and progression. However, p190B transgene expression in the developing mammary gland also inhibited signaling to AKT and ERK suggesting that similar signaling pathways are altered downstream of exogenous p190B expression during mammary gland development and tumorigenesis [14]. In the p190B transgenic tumors the expression levels of PAK and ROCK proteins were only modestly decreased. Furthermore, phospho-ROCK was elevated in p190B transgenic tumors suggesting that despite effects on total protein levels, signaling through ROCK may still be important for MMTV-Neu induced tumorigenesis.

The Rho signaling network is highly complex, and it is likely that multiple signaling pathways converge to modulate PAK, ROCK, AKT, and ERK. For example, ErbB2 and ErbB3 cooperate to promote MMTV-Neu induced tumorigenesis, and activation of AKT and MAPK downstream of ErbB2/ErbB3 affects tumor cell proliferation [21]. Interestingly, by Western analysis we detected a slight decrease in expression of the full-length form of ErbB3 concomitant with the appearance of a lower molecular weight form suggesting that ErbB3 may be proteolytically cleaved in the p190B transgenic tumors. Consistent with this possibility we detected an increase in MMP3 expression in the p190B transgenic tumors. Furthermore, MMP-dependent cleavage of the related ErbB4 produces a cytoplasmic fragment that regulates tumor cell proliferation [47]. It is possible that decreased expression of full length ErbB3 at the cell surface is responsible for the diminished activation of the downstream effectors AKT and ERK in the tumors expressing exogenous p190B. Future studies will be aimed at elucidating how these complex signaling interactions regulate MEC morphogenesis and invasion utilizing primary MECs in the 3 D culture system.

To reconcile the apparent inconsistencies between the pro-tumorigenic effects of exogenous p190B expression and increased Rac1 activity with the diminished signaling to the downstream effectors we considered the possibility that ROS were elevated downstream of p190B/Rac1. ROS have been shown to act downstream of Rac1 to facilitate transformation and metastasis [15,28,48], and in the presence of cellular stress, ROS promoted oxidative stress, diminished ERK phosphorylation, and cell death [49]. Because of the lack of an *in situ *assay to detect ROS in the tumor tissue, we examined protein carbonylation as an indicator of oxidative stress. Interestingly, protein carbonylation is elevated suggesting that oxidative stress may be increased in p190B transgenic tumors. Expression of matrix metalloproteases (MMPs) is also regulated by ROS [29,50,51], and MMP-3 was upregulated in the p190B transgenic tumors as another potential indicator of an increase in ROS downstream of elevated Rac1 in the tumors expressing exogenous p190B. We hypothesize that p190B may promote tumor formation and progression in part by affecting Rac/ROS mediated signaling, however, additional studies will be required to prove this hypothesis.

Rho GTPases including RhoA, Rac1, and Cdc42 are elevated and hyperactivated in breast cancer [4,5,32]. We, therefore, might have anticipated that exogenous expression of p190B, an inhibitor of Rho GTPases, would have tumor suppressor functions. However, our loss and gain of function studies indicate that p190B has pro-tumorigenic actions and that it may facilitate tumor formation and progression in part by modulating Rac1 activity. These results suggest that regulation of Rho GTPase activity *in vivo *is likely more complex than what occurs in cultured cells.

Several lines of evidence suggest that the closely related p190A RhoGAP functions as a tumor suppressor. For example, p190A is located on chromosome 19q13.3, which is deleted in human gliomas [52]. P190A also inhibits Ras-induced transformation in fibroblasts [53] and suppresses platelet derived growth factor (PDGF) induced glioma formation in mice [54]. Thus, these two related RhoGAPs may have distinct actions during tumorigenesis.

## Conclusions

P190B RhoGAP, a major inhibitor of the Rho GTPases *in vitro*, has pro-tumorigenic functions during mammary tumor formation and progression. P190B may mediate its effects on mammary tumorigenesis in part by affecting Rac1 activity. Rho GTPases are overexpressed and hyperactivated in human breast cancers, and a number of *in vitro *and limited *in vivo *studies indicate that they function as oncogenes during tumor progression. Thus, it was intriguing that an inhibitor of the Rho GTPases has pro-tumorigenic functions during mammary tumor formation and progression. These studies demonstrate the importance of investigating the functions of this complex signaling network *in vivo *during the stochastic process of tumor formation and progression.

## Abbreviations

Akt: protein kinase B; Bad: Bcl-2/Bcl-XL-associated death promoter; BrdU: bromodeoxyuridine; Cdc42: cell division cycle 42; ECM: extracellular matrix; ErbB2: erythroblastosis B2; ErbB3: erythroblastosis B3; ERK: extracellular regulated kinase; ERM: ezrin/radixin/moesin; GAP: GTPase activating protein; GEF: guanine nucleotide exchange factor; GTP: guanine triphosphatase; IRES: internal ribosomal entry site; MEC: mammary epithelial cell; MG: mammary gland; MMP: matrix metalloprotease; MMTV: mouse mammary tumor virus; Neu: rat homolog of ErbB2; PAK: P21 activated kinase; PDGF: platelet derived growth factor; Rac: Ras-related C3 botulinum toxin substrate 1; Rho: Ras homologous; ROCK: Rho associated kinase; ROS: Reactive oxygen species; rtTA: reverse tetracycline transactivator; TetO: tetracycline operator.

## Competing interests

The authors declare that they have no competing interests.

## Authors' contributions

PM performed GTPase assays, Western blotting, 3 D culture invasion assays, assisted with adhesion assays, interpreted data, and assisted with manuscript preparation. JS cut tissue sections, performed and quantified immunostaining, interpreted data, and did luciferase assays. MH monitored mice for tumor formation, dissected tumors, quantified metastasis, and analyzed data. PC performed adhesion assays, inhibitor experiments, quantified 3 D culture data, and analyzed data. MR quantified metastasis and assisted with data analysis. BH-S monitored mice for tumor formation, dissected tumors, and contributed to the study design. EG and LC provided the MMTV-rtTA transgenic mice and the plasmid construct used to create the Tet-O-p190B transgenic mice. SGH performed statistical analysis. JR co-directed the design and implementation of the study and interpreted data. TV-G co-directed the design and implementation of the study, obtained cohorts of mice for study, performed 3 D culture experiments, interpreted data, and drafted the manuscript. All authors read and approved the final manuscript.

## Supplementary Material

Additional file 1 **Supplemental figure 1. Rac and ERK inhibition disrupt polarity and lumen formation and increase invasion of primary p190B transgenic and control MEC acini in 3 D culture**. **(a) **Representative images of phenotypes detected in DMSO control, Rac, and ERK inhibitor treated 3 D cultures of primary rtTA control and p190B MECs. Gree*n *=αtubulin, Red = pERM (apical polarity marker), and Blue = nuclei. Arrows indicate invasion into the surrounding matrix. Size bars represent 10 μm. **(b) **Quantification of the average number of invadipodia in vehicle, Rac inhibitor, and ERK inhibitor treated 3 D cultures (100 structures per experiment were quantified and data represent *n *=3 experiments) is graphed. Exogenous p190B expression resulted in a statistically significant increase in invasion in DMSO vehicle treated cultures (*P *= 0.034). Inhibition of Rac and ERK resulted in statistically significant increases in invasion in control rtTA (*P *= 0.006 and *P *= 0.007, respectively) and in p190B (*P *= 0.047 and *P *= 0.039, respectively) mammary acini. Statistically significant differences were not detected between genotypes in Rac or ERK inhibitor treated cultures. T tests were used to compare means and error bars represent standard error of the mean. **(c) **Quantification of the percentage of structures with disrupted lumen formation in DMSO vehicle, Rac inhibitor, and ERK inhibitor treated 3 D cultures (100 structures per experiment were quantified and data represent *n *=3 experiments) is graphed. Exogenous p190B did not have a statistically significant affect on lumen formation in DMSO vehicle treated cultures (*P *> 0.05). Inhibition of Rac and ERK resulted in statistically significant differences in disruption of lumen formation in control rtTA mammary acini (*P *= 0.017 and *P *= 0.001, respectively). Disruption of lumen formation was statistically significantly altered in p190B transgenic mammary acini treated with the ERK inhibitor (*P *= 0.001), but not when treated with the Rac inhibitor (*P *> 0.05). Statistically significant differences were not detected between genotypes in Rac or ERK inhibitor treated cultures. T tests were used to compare means and error bars represent standard error of the mean. Click here for file
