# Supplementary material for: P190B RhoGAP has pro-tumorigenic functions during MMTV-Neu mammary tumorigenesis and metastasis
Source: Breast Cancer Res. 2010 Sep 22;12(5):R73. doi: 10.1186/bcr2643 (PMC3096962; doi:10.1186/bcr2643)
Supplement: Additional file 1 — Supplemental figure 1. Rac and ERK inhibition disrupt polarity and lumen formation and increase invasion of primary p190B transgenic and control MEC acini in 3 D culture. (a) Representative images of phenotypes detected in DMSO control, Rac, and ERK inhibitor treated 3 D cultures of primary rtTA control and p190B MECs. Green =αtubulin, Red = pERM (apical polarity marker), and Blue = nuclei. Arrows indicate invasion into the surrounding matrix. Size bars represent 10 μm. (b) Quantification of the average number of invadipodia in vehicle, Rac inhibitor, and ERK inhibitor treated 3 D cultures (100 structures per experiment were quantified and data represent n =3 experiments) is graphed. Exogenous p190B expression resulted in a statistically significant increase in invasion in DMSO vehicle treated cultures (P = 0.034). Inhibition of Rac and ERK resulted in statistically significant increases in invasion in control rtTA (P = 0.006 and P = 0.007, respectively) and in p190B (P = 0.047 and P = 0.039, respectively) mammary acini. Statistically significant differences were not detected between genotypes in Rac or ERK inhibitor treated cultures. T tests were used to compare means and error bars represent standard error of the mean. (c) Quantification of the percentage of structures with disrupted lumen formation in DMSO vehicle, Rac inhibitor, and ERK inhibitor treated 3 D cultures (100 structures per experiment were quantified and data represent n =3 experiments) is graphed. Exogenous p190B did not have a statistically significant affect on lumen formation in DMSO vehicle treated cultures (P > 0.05). Inhibition of Rac and ERK resulted in statistically significant differences in disruption of lumen formation in control rtTA mammary acini (P = 0.017 and P = 0.001, respectively). Disruption of lumen formation was statistically significantly altered in p190B transgenic mammary acini treated with the ERK inhibitor (P = 0.001), but not when treated with the Rac inhibitor (P > 0.05) [file bcr2643-S1.PPT]

## Slide 1
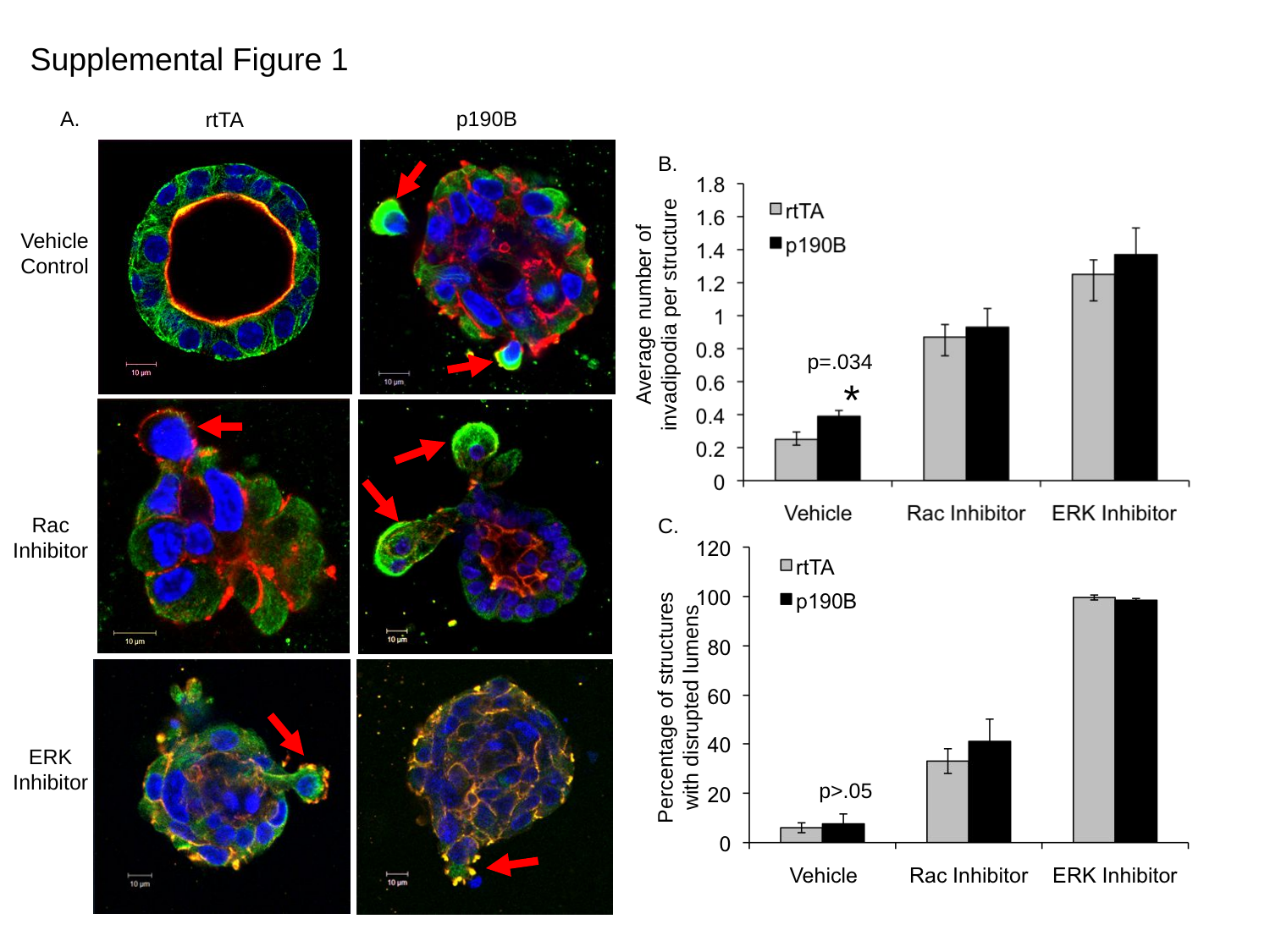

E.
Average number of invadipodia per structure
p=.034
 *
Supplemental Figure 1
A.
p190B
rtTA
B.
Vehicle
Control
Rac Inhibitor
C.
Percentage of structures with disrupted lumens
p>.05
ERK Inhibitor
